# Antiplatelet and Antithrombotic Activity of a Traditional Medicine, Hwangryunhaedok-Tang

**DOI:** 10.3389/fphar.2018.01502

**Published:** 2019-01-09

**Authors:** Kyungho Kim, Hyun Ju Do, Tae Woo Oh, Kwang-Youn Kim, Tae Hoon Kim, Jin Yel Ma, Kwang-Il Park

**Affiliations:** ^1^Korean Medicine-Application Center, Korea Institute of Oriental Medicine, Daegu, South Korea; ^2^Department of Food Science and Biotechnology, Daegu University, Gyeongsan, South Korea

**Keywords:** platelet, Hwangryunhaedok-tang (Hwanglianjiedu-tang), thrombosis, platelet aggregation and activation, hemostasis

## Abstract

Platelet activation and accumulation at the site of vascular injury are central to thrombus formation resulted in thrombotic disorders. Medicinal herbs could be one of the most important pharmaceutical agents that ameliorate thrombotic disorders, such as unstable angina, myocardial infarction, stroke, and peripheral vascular diseases. Hwangryunhaedok-tang (HRT) is a traditional herbal medicine that displays multiple biological properties including anti-inflammatory abilities. However, its role in platelet activation has not been fully studied. Hence, we examined whether HRT has a potent inhibitory effect on platelet aggregation and thrombus formation. We demonstrated that HRT (30, 50, and 100 μg/ml) significantly impaired thrombin- and collagen-related peptide-induced platelet aggregation, granule secretion, thromboxane B_2_ generation, and intracellular Ca^2+^ mobilization. Biochemical studies revealed that HRT is involved in inhibiting the phosphorylation of phospholipase C and protein kinase B. The oral administration of HRT (30, 50, and 100 mg/kg once daily for 1 and/or 7 days) efficiently ameliorates ferric chloride induced arterial thrombus formation *in vivo*. Tail bleeding time was not significantly increased. The qualitative phytochemical constituents of the HRT extract were investigated using high-performance liquid chromatography. Our results demonstrated that HRT shows potential antiplatelet and antithrombotic effects without affecting hemostasis. Hence, HRT could be an effective therapeutic agent for the treatment of thrombotic diseases.

## Introduction

Platelets play an important role in both thrombosis and hemostasis. Platelet adhesion and activation at the site of activated endothelial cells and/or subendothelial matrix proteins such as collagen and von Willebrand factor are the first steps in hemostasis (Nieswandt et al., 2002). The adherent and activated platelets induce the increase in intracellular Ca^2+^ concentration and protein kinase activation through a distinct signaling pathway. The activated platelets secrete thromboxane A_2_ and granular contents, such as ADP, serotonin, and growth factors, which further activate other platelets and facilitate platelet aggregation to prevent blood loss (Varga-Szabo et al., 2009; Li et al., 2010; Bye et al., 2016). However, in pathological condition, unwanted platelet activation leads to increased platelet aggregation and pathologic thrombosis, which is a primary factor in the development of thrombotic disorders, including unstable angina, myocardial infarction, ischemic stroke, and peripheral vascular diseases (Davies and Thomas, 1985; Genton et al., 1986; Trip et al., 1990). Consequently, understanding the mechanism underlying platelet activation and aggregation that precisely inhibits platelet functions is important as a promising approach for the treatment of thrombotic diseases.

Traditional herbal medicines are plant-derived substances that have been used to treat illnesses in combinatory therapeutic strategies over 1000s of years. A combination of different types of herbs is often used to increase therapeutic efficacy and lower toxicity. Hwangryunhaedok-tang (HRT), also known as Huang-Lian-Jie-Du-Tang in China, is a traditional herbal medicine that consists of four different herbs: cortex phellodendri (Phellodendron amurense Rupr, Rutaceae), Radix scutellariae (*Scutellaria baicalensis* Georgi, Labiatae), Rhizoma coptidis (*Coptis chinensis* Franch, Ranunculaceae), and Fructus gardenia (Gardenia jasminoides Ellis, Rubiaceae). HRT has primarily been used to treat inflammatory-related diseases, such as gastritis, dermatitis, and hepatitis, for a long time in Asia (Tie et al., 2000). Recently, the pharmacological effects of HRT revealed that the inflammatory responses induced by the carrageenan-injected air pouches model were significantly reduced by the suppression of eicosanoid and nitric oxide production (Zeng et al., 2011). Further, treatment with HRT showed therapeutic efficacy in various inflammatory conditions, such as dextran sulfate sodium-induced colitis in mice and collagen-induced arthritis in rats (Tie et al., 2000; Yue et al., 2013a,b). However, no studies have yet examined whether HRT has a potent inhibitory effect on platelet activation and thrombosis.

In this study, we demonstrate that HRT plays a pivotal role in platelet activation and thrombus formation. HRT is important for platelet activation and aggregation induced by numerous agonists *in vitro*. Biochemical study revealed that HRT regulates the phosphorylation of phospholipase C (PLC) and protein kinase B (AKT) during cell activation. Further, *in vivo* studies revealed that the oral administration of HRT efficiently ameliorates ferric chloride (FeCl_3_)-induced arterial thrombus formation without prolonging tail bleeding time. Our studies provide evidence that the prescription of HRT could be an important therapeutic approach for regulating thrombotic diseases.

## Materials and Methods

### Animals

Wild-type (WT) (C57BL/6, 6 weeks old) mice were obtained from the Samtako Laboratory Animal Center (Suwon, South Korea) and acclimated for 1 week. The mice were housed in a conventional animal facility with free access to food and water in a controlled temperature and humidity environment under a 12:12 h light–dark schedule. The animals were cared for in accordance with the dictates of the National Animal Welfare Law of Korea. The animal experiments (reference numbers #D-18-009) approved by the Animal Care and Use Committee of the Korea Institute of Oriental Medicine (KIOM, Daegu, South Korea) were performed in accordance with their guidelines of the Animal Management Committee of the Korea Institute of Oriental Medicine.

### Reagents

Human thrombin, PGE1, dimethyl sulfoxide (DMSO), ADP, fibrinogen, TXA_2_ analog U46619, ferric chloride (FeCl_3_), and all the reagents were purchased from Sigma (St. Louis, MO, United States). D-Phe-Pro-Arg-chloromethyl ketone (PPACK) was purchased from EMD Millipore (Billerica, MA, United States). Collagen-related peptide (CRP) was obtained from Dr. Richard Farndale (Department of Biochemistry, University of Cambridge, United Kingdom). Phycoerythrin (PE)-conjugated isotype control IgGs, rat monoclonal antibodies against mouse P-selectin, and activated αIIbβ3 (JON/A) were obtained from Emfret Analytics (Eibelstadt, Germany). Antibodies against phospho-PLCγ2 at Tyr759, phospho-PLCβ3 at Ser1105, phospho-Akt at Ser473, Total Akt, Total PLCγ2, Total PLCβ3, and actin were obtained from Cell Signaling (Danvers, MA, United States). Calcium dye (FLIPR Calcium Assay kit) was obtained from Molecular Devices (Sunnyvale, CA, United States).

### Hwangryunhaedok-Tang (HRT) Preparation

Hwangryunhaedok-tang was obtained from the Yeongcheon Oriental Herbal Market (Yeongcheon, South Korea). The mixture of dried Rhizoma coptidis (Ranunculaceae) (250 g), Radix scutellariae (Labiatae) (250 g), Cortex phellodendri (Rutaceae) (250 g), and Fructus gardenia (Rubiaceae) (250 g) were placed in 10,000 ml of distilled water and heated at 115°C in an extractor (Gyeongseo Extractor Cosmos-600, Inchon, South Korea) for 3 h. The resulting extract was filtered using a standard test sieve (150 μm) (Retsch, Hann, Germany) and was freeze-dried for a yield rate of 39%. The lyophilized HRT powder was dissolved in 0.01% DMSO to yield a final concentration of 10 mg/ml.

### HPLC Analysis

High-performance liquid chromatography (HPLC) (Shimadzu, Tokyo, Japan) combined with a photodiode array (PDA) detector was used for the chromatographic analysis of HRT. The HPLC analysis was performed using a YMC-Pack ODS A-302 column (4.6 mm i.d. × 150 mm; YMC, Co., Kyoto, Japan), and the solvent system consisted of a gradient mode with an initial 10% CH_3_CN with 10 mM phosphate buffer increased to CH_3_CN over 120 min (temperature: 40°C; flow rate: 1.0 mL/min; UV detection; 280 nm). Successive column chromatography was conducted using YMC GEL ODS AQ 120-50S (YMC, Co., Kyoto, Japan). Quantification of the five standard compounds was carried out by HPLC analysis using the external standard method by constructing standard curves. Five concentrations were used for preparation of the calibration curve and the calibration curve of pure solutions of the selected compounds was completely linear (*R*^2^ > 0.999). Five concentrations were used for preparation of the calibration curve and the calibration curve of pure solutions of the all standard compounds was completely linear (*R*^2^ > 0.999). The retention times of geniposide (**1**) (*t*_R_ 5.3 min), coptisine (**2**) (*t*_R_ 17.9 min), palmatine (**3**) (*t*_R_ 35.5 min), berberine (**4**) (*t*_R_ 32.2 min), and baicalin (**5**) (*t*_R_ 45.1 min) were then detected at 240 and 277 nm.

### Platelet Preparation

Mouse platelets were isolated as previously described (Kim et al., 2013). Briefly, citrate-dextrose solution (ACD, Sigma) treated mouse blood was drawn from WT (6–8 weeks old) mice. Whole blood was centrifuged at 300 *g* for 20 min at room temperature to obtain platelet-rich plasma (PRP). The PRP was collected and re-centrifuged at 700 *g* for 4 min in the presence of 0.5 μM PGE1. The platelet pellet was suspended in HEPES-Tyrode buffer (5 mM HEPES/NaOH, pH 7.3, 5 mM glucose, 136 mM NaCl, 12 mM NaHCO_3_, 2.7 mM KCl) containing 10% ACD, and centrifuged at 700 *g* for 5 min. The pellet was re-suspended in HEPES-Tyrode buffer, and the final suspensions were adjusted to 3 × 10^8^ platelets/ml.

### Platelet Aggregation Assay

Platelet aggregation assay was performed as previously described (Kim et al., 2017). Washed platelets in modified HEPES-Tyrode buffer were pre-incubated with 0.01% DMSO or various concentrations of HRT (30, 50, and 100 μg/ml) for 10 min for at 37°C and then stimulated with numerous agonists. Platelet aggregation was monitored in a platelet aggregometer (Chronolog, Corp., Havertown, PA, United States) at 37°C with stirring (1,000 rpm).

### ATP Release Assay

Washed platelets were pre-incubated with 0.01% DMSO or various concentrations of HRT (30, 50, and 100 μg/ml) for 10 min at 37°C and then stimulated with Thrombin and CRP in a platelet aggregometer at 37°C with stirring (1,000 rpm). The reaction was terminated by centrifugation and the supernatants were collected and used for the assay. Adenosine triphosphate (ATP) release was measured in a luminometer (Glomax Explorer multimode microplate reader, Promega) using an ATP assay kit (Biomedical Research Service Center, Buffalo, NY, United States).

### TXB_2_ Generation Assay

Washed platelets were pre-incubated with 0.01% DMSO or various concentrations of HRT (30, 50, and 100 μg/ml) for 10 min at 37°C and then stimulated with thrombin (0.05 U/ml) and CRP (0.5 μg/ml) in an aggregometor at 37°C with stirring (1,000 rpm). The reaction was stopped after 5 min by the addition of 2 mM EGTA containing 0.1 M KCl and 5 mM indomethacin for 10 min on ice. The mixture was then centrifuged at 6,000 *g* for 3 min, and the supernatant was stored at -80°C until analysis. Thromboxane B_2_ (TXB_2_) levels were measured using an enzyme-linked immunosorbent assay kit (Enzo life Sciences, Farmingdale, NY, United States) according to the manufacturer’s protocol.

### Flow Cytometric Analysis

Washed platelets were pre-incubated with 0.01% DMSO or various concentrations of HRT (30, 50, and 100 μg/ml) for 10 min at 37°C. Platelets were treated with 0.05 U/ml thrombin or 0.5 μg/ml CRP for 5 min at 37°C, followed by incubation with PE-conjugated antibodies against P-selectin or activated αIIbβ3 integrin (JON/A) for 15 min. Cells were analyzed by flow cytometry (Gallios, Beckman Coulter).

### Ca^2+^ Mobilization

Ca^2+^ mobilization was measured as previously described (Kim et al., 2015). Platelets (1 × 10^8^/ml) were suspended in HEPES-Tyrode buffer, pH 7.4 without CaCl_2_ and treated with 0.01% DMSO or HRT (30, 50, 100 μg/ml) for 10 min at 37°C. Cells were incubated with a Ca^2+^ dye (FLIPR Calcium 5 Assay kit) for 30 min at 37°C in the dark, followed by stimulation with thrombin (0.05 U/ml) or CRP (0.5 μg/ml). Cytosolic Ca^2+^ levels were measured using a spectrofluorometer (Spectramax I3, Molecular Devices) with an excitation wavelength of 485 nm and an emission wavelength of 525 nm. Ca^2+^ mobilization was quantified by area under the curve (AUC) and expressed in relative fluorescence units.

### Immunoblotting

Mouse platelets were stimulated by thrombin (0.05 U/ml) or CRP (0.5 μg/ml) in the presence or absence of three different concentrations of HRT under stirring conditions (1,000 rpm) in an aggregometer. To measure the phosphorylation levels of the kinases, platelets (6 × 10^8^ platelets/ml) were lysed in an equal volume of 2x ice-cold lysis buffer (TBS, pH 7.4, containing 2% Triton X-100, 0.1% SDS, 2 mM EDTA, 2 mM Na_3_VO_4_, phosphatase inhibitor cocktail, protease inhibitor cocktail, and 2 mM phenylmethylsulfonyl fluoride) and sonicated. An equal amount of protein (30 μg) was electrophoresed under reduced conditions and was immunoblotted, followed by reprobing with different antibodies. The band density was measured by densitometry using Image J (v1.50b). The phosphorylation level of the kinases was calculated by normalization of the density of antibodies against the phosphorylated kinases to that of the antibodies against total kinases.

### FeCl_3_-Induced *in vivo* Thrombosis

Mice were orally administered with 0.5% low viscosity of CMC and/or HRT (100 mg/kg) or ASA (50 mg/kg) once a day for 1 day and/or for 7 days. The mice were anesthetized with isoflurane 2 h after the last administration. The left carotid artery was isolated, and then a filter paper (2 mm diameter) soaked with 10% (460 mM) ferric chloride (FeCl_3_) was placed on top of the artery for 2 min. Blood flow was then monitored until 10 min after blood occlusion using a blood flowmeter (AD instruments, Blood flowmeter).

### Tail Bleeding Time

Mice were orally administered with 0.5% low viscosity of CMC and/or HRT (100 mg/kg) or ASA (50 mg/kg) once a day for 1 day and/or for 7 days. The mice were anesthetized with a 2% isoflurane in oxygen mixture 2 h after the last administration. The body temperature was maintained at 37°C using a heating pad. Using a sharp razor blade, 5 mm of the tail was removed and the tail held in a 15 ml tube containing 13 ml of PBS prewarmed to 37°C. Tail bleeding was monitored and time to cessation of blood flow was measured, and after 10 min the bleeding was stopped by cauterization.

### Statistical Analysis

Data analysis was performed using GraphPad Prism 5. Statistical significance was assessed by ANOVA and Tukey’s test or Dunnett’s test for comparison of multiple groups or Student’s *t-*test for comparison of two groups. A *P*-value less than 0.05 was considered significant.

## Results

### HRT Regulates Agonist-Induced Platelet Aggregation and ATP Secretion

**FIGURE 1:**
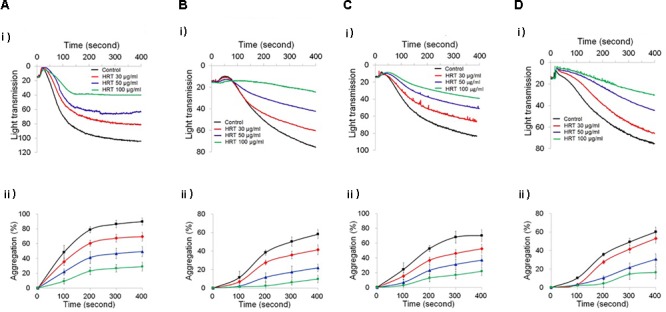
Inhibitory effect of Hwangryunhaedok-tang (HRT) on platelet aggregation following stimulation with various agonists. Washed platelets were preincubated with various concentrations of HRT (30, 50, and 100 μg/ml) for 10 min at 37°C and then stimulated with 0.05 U/ml Thrombin **(A)**, 0.5 μg/ml CRP **(B)**, 10 μM ADP **(C)**, and 1 μM U46619 **(D)**. In the ADP-induced aggregation assay, 30 μg/ml of human FG was added to the platelet suspension before ADP stimulation. (i) Platelet aggregation and (ii) quantitative graphs. Data represent the mean ± SD (*n* = 3).

**FIGURE 2:**
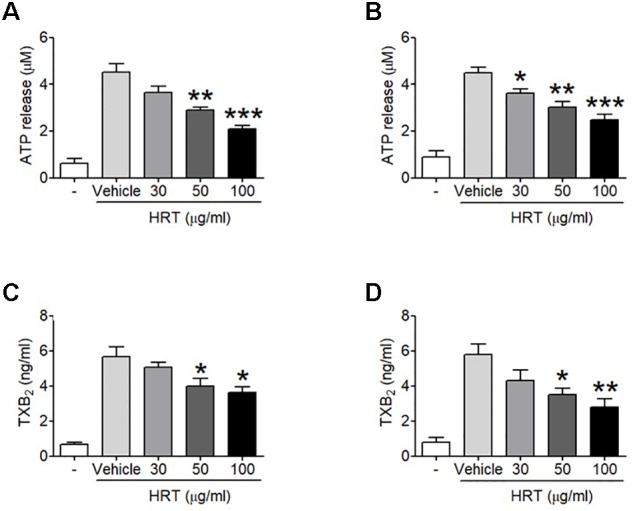
Inhibitory effect of HRT on ATP secretion and TXB2 generation following stimulation with thrombin and CRP. Washed platelets were preincubated with various concentration of HRT (30, 50, and 100 μg/ml) for 10 min at 37°C before adding a luciferin/luciferase reagent. After the luciferin/luciferase reagent added, platelets were stimulated with 0.05 U/ml thrombin **(A)** or 0.5 μg/ml CRP **(B)**. ATP secretion was measured using a luminometer. Effect of HRT on TXB2 generation was measured using a TXB2 ELISA assay kit. Washed platelet were pretreated with various concentration of HRT (30, 50, and 100 μg/ml) for 10 min, then stimulated with 0.05 U/ml thrombin **(C)** or 0.5 μg/ml CRP **(D)**. Data represent the mean ± SD (*n* = 3). ^∗^*P* < 0.05, ^∗∗^*P* < 0.01, and ^∗∗∗^*P* < 0.001 versus vehicle control after ANOVA and Dunnett’s test.

To study the role of HRT on platelet function, we first examined platelet aggregation induced by thrombin (0.05 U/ml) and CRP (0.5 μg/ml) stimulation (Figures 1A,B). We observed that compared to the vehicle control, HRT exhibited significantly decreased platelet aggregation in a concentration-dependent manner (30, 50, and 100 μg/ml HRT), but not by 10 μg/ml HRT (data not shown). Aggregation induced by ADP (10 μM) and U46619 (1 μM) were also defective at concentrations of 30, 50, and 100 μg/ml HRT compared to the vehicle control (Figures 1C,D). The reduced platelet aggregation of HRT-incubated platelets (100 μg/ml) was nullified at high concentrations of agonists (>0.2 U/ml thrombin, >2 μg/ml CRP, >30 μM ADP, and >10 μM U46619) (Supplementary Figure S1). Compared to the vehicle control, HRT treatment (30, 50, and 100 μg/ml) in the ATP secretion assays showed significant defects in ATP secretion induced by thrombin (0.05 U/ml) and CRP (0.5 μg/ml) (Figures 2A,B). We also assessed TXB_2_ generation after stimulation thrombin (0.05 U/ml) and CRP (0.5 μg/ml). TXB_2_ was dramatically elevated by both agonists, but HRT showed a potent inhibition of thrombin and CRP-induced TXB_2_ formation in a concentration-dependent manner (Figures 2C,D). These results suggest that HRT plays an important role in stimulating platelet aggregation, ATP secretion, and TXB_2_ generation.

### HRT Plays an Important Role in Regulating Platelet Activation

**FIGURE 3:**
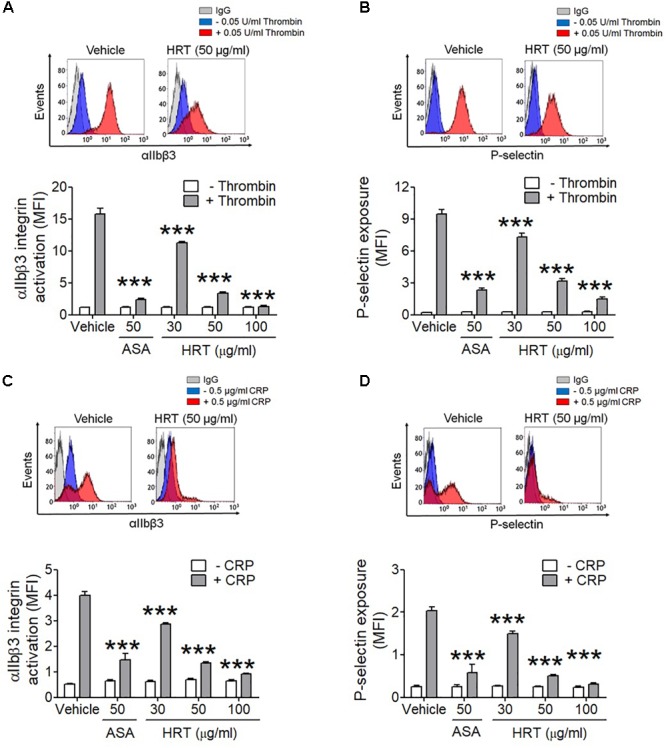
Inhibitory effect of HRT on αIIbβ3 integrin activation and P-selectin exposure during platelet activation. Mouse platelets were pre-treated with various concentration of HRT (30, 50, and 100 μg/ml) or ASA, 50 μg/ml, and stimulated with 0.05 U/ml thrombin **(A,B)** or 0.5 μg/ml CRP **(C,D)**. αIIbβ3 integrin activation and P-selectin exposure were analyzed by flow cytometry as described in Section “Materials and Methods.” Binding of anti-activated αIIbβ3 (JON/A) and anti-P-selectin antibodies to platelets was calculated by the ratio of the geometric mean fluorescence intensity (MFI) value of antibodies to that of control IgG. Data represent mean ± SD (*n* = 3). ^∗∗∗^*P* < 0.001 vs. vehicle control after ANOVA and Dunnett’s test.

We further examined whether HRT regulates P-selectin exposure and αIIbβ3 integrin activation during cell activation. HRT (30, 50, and 100 μg/ml HRT) significantly inhibited P-selectin exposure and αIIbβ3 integrin activation in response to thrombin (0.05 U/ml) (Figures 3A,B) and CRP (0.5 μg/ml) (Figures 3C,D) stimulation in a dose dependent manner. These results suggest that HRT plays an important role in regulating platelet activation.

### Inhibitory Effect of HRT on Ca^2+^ Mobilization

**FIGURE 4:**
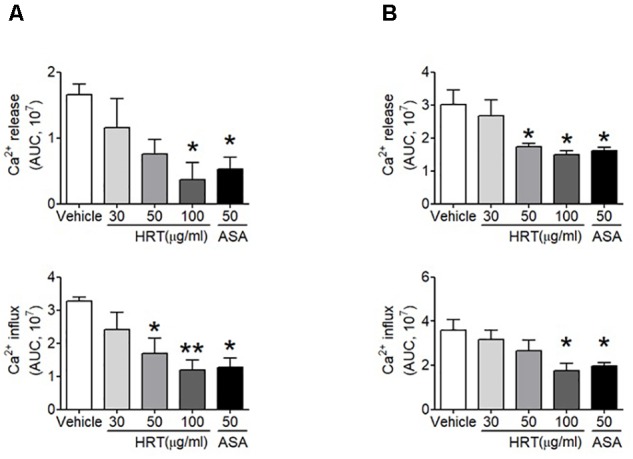
Hwangryunhaedok-tang is important for platelet activation through Ca^2^x^+^ mobilization. Mouse platelets were resuspended in HEPES-Tyrode buffer without 1 mM CaCl_2_ and preincubated with various concentration of HRT (30, 50, and 100 μg/ml) or ASA, 50 μg/ml, and then incubated with a calcium-sensitive dye for 30 min at 37°C in the dark. After treatment with a Ca^2+^ dye, platelets were stimulated with 0.05 U/ml thrombin **(A)** or 0.5 μg/ml CRP **(B)** for 10 min and 2 mM CaCl_2_ was then added. Intracellular Ca^2+^ release and influx were measured and quantified by the AUC (arbitrary units). Quantitative data represent the mean ± SD (*n* = 3). ^∗^*P* < 0.05 and ^∗∗^*P* < 0.01 versus vehicle control after ANOVA and Dunnett’s test.

To investigate the mechanism by which HRT treatment contributes to platelet activation, we further examined whether HRT is important for the agonist-induced elevation of intracellular Ca^2+^ mobilization, which is a key step in platelet activation. We observed that intracellular Ca^2+^ release and influx were significantly increased with thrombin and CRP stimulation (Figures 4A,B), whereas Ca^2+^ mobilization was inhibited in a dose dependent manner when platelets were treated with HRT (30, 50, and 100 μg/ml) prior to thrombin or CRP stimulation (Figures 4A,B). These results suggest that HRT regulates thrombin- or CRP-induced Ca^2+^ mobilization during cell activation.

### HRT Plays an Important Role in Regulating the Phosphorylation of PLC and AKT

**FIGURE 5:**
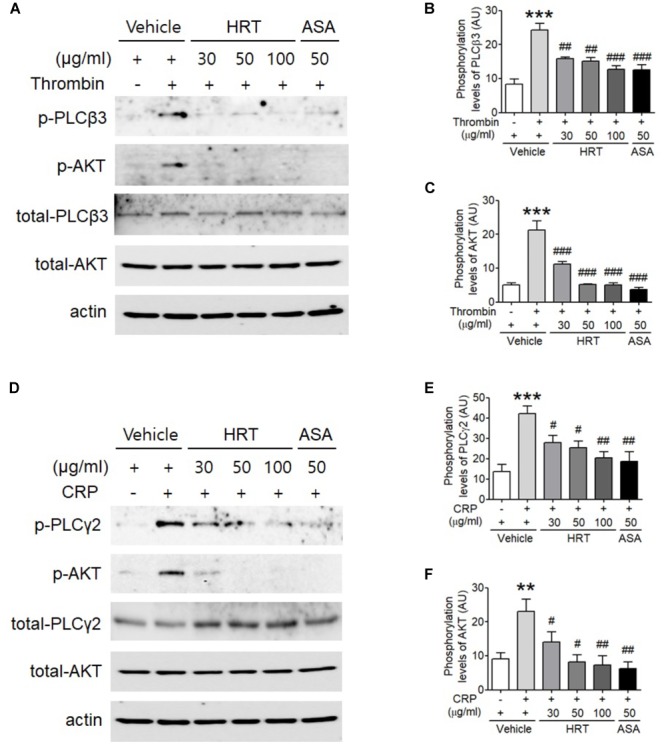
Hwangryunhaedok-tang is important for regulating the phosphorylation of PLC and AKT. Mouse platelets were pre-treated with various concentration of HRT (30, 50, and 100 μg/ml) or ASA, 50 μg/ml, and stimulated with 0.05 U/ml thrombin **(A)** or 0.5 μg/ml CRP **(D)**. Equal amounts (30 μg) of cell lysate protein were immunoblotted to determine specific inhibition of PLC and AKT phosphorylation. Representative blots **(A,D)**. Quantitative graphs **(B,C,E,F)**. Data represent the mean ± SD (*n* = 3–4). ^∗∗∗^*P* < 0.001 vehicle control (stimulated) versus vehicle control (unstimulated) after Student’s *t*-test and ^###^*P* < 0.001 (HRT and/or ASA treated samples) versus vehicle control (stimulated) after ANOVA and Turkey’s test.

The binding of agonists to G-protein-coupled receptors is involved in the activation of Ca^2+^ signaling via the phosphorylation of PLC-AKT signaling (Xiang et al., 2012), thereby regulating platelet activation. Since the treatment of HRT is critical for Ca^2+^ mobilization during cell activation, we examined whether HRT treatment regulates the phosphorylation of PLC and its downstream kinase, AKT. We observed that compared to the controls, HRT treatment exhibited a significant decrease in the phosphorylation of PLCβ3 and AKT following thrombin stimulation (Figures 5A–C) and in the phosphorylation of PLCγ2 and AKT following CRP stimulation (Figures 5D–F). These results suggest that HRT plays an important role in the phosphorylation of PLC and AKT and is not selective to specific signal transduction pathways.

### HPLC Analysis of HRT

The chromatograms of reference compounds including geniposide, coptisine, berberine, palmatine, and baicalin (Figures 7A,B) and an HRT sample of five major constituents (Figures 7C,D) were simultaneously determined with HPLC. The retention times (geniposide: 5.3, coptisine: 17.9, berberine: 32.2, palmatine: 35.5, and baicalin: 45.1 min) and contents (5.3–29.9 mg/g) of the five major constituents are summarized in Table 1.

**Table 1:** Quantitative analysis of the five major constituents in HRT.

		Regression equation^a^	Linear range	Content^b^
Compounds	*t*_R_ (min)	(*Y* = aX + b, *R*^2^)	(mg/mL)	(mg/g)
Geniposide	5.3	Y = 0.012X + 35.9, 0.9999	50–500	19.7 ± 0.5
Coptisine	17.9	Y = 0.082X - 14.9, 0.9998	50–500	5.3 ± 0.2
Berberine	32.2	Y = 0.054X + 10.8, 0.9995	50–500	22.2 ± 0.7
Palmatine	35.5	Y = 0.051X + 20.0, 0.9997	50–500	9.2 ± 0.3
Baicalin	45.1	Y = 0.068X - 21.7, 0.9995	50–500	29.9 ± 0.7

*^*a*^*Y* = peak area and *X* = concentration. ^*b*^Mean ± SD (*n* = 3).*

### HRT Inhibits *in vivo* Thrombosis but Not Hemostasis

**FIGURE 6:**
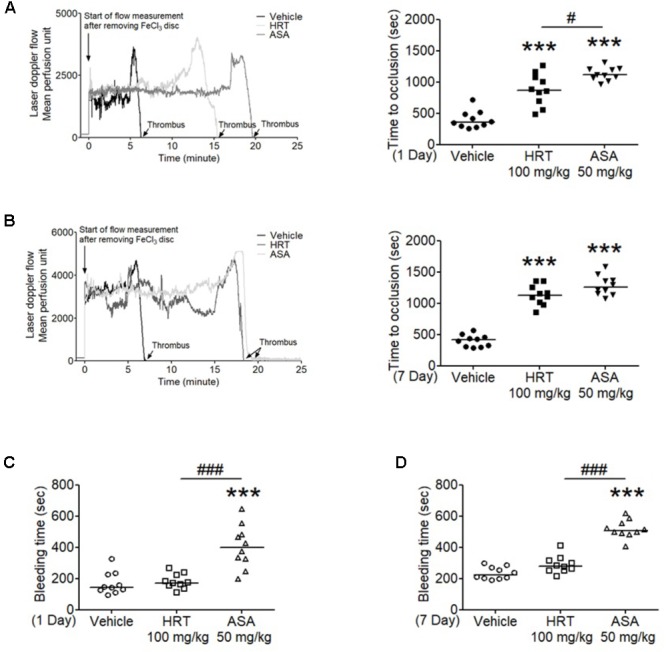
Hwangryunhaedok-tang delayed FeCl_3_-induced arterial thrombus formation but not hemostasis. FeCl_3_-induced arterial thrombus formation was performed as described in Section “Materials and Methods.” After oral administration of HRT and ASA for 1 day **(A)** or 7 days **(B)**, the mouse carotid artery was treated with 10% FeCl_3_ for 2 min, and blood flow traces were monitored until stable occlusion took place. Horizontal bars represent the median occlusion time (*n* = 10). After oral administration of HRT and ASA for 1 day **(C)** or 7 days **(D)**, tails of vehicle (open circle), HRT (open square), and ASA (open triangle) treated mice were amputated, and bleeding time was monitored as described in Section “Materials and Methods.” Horizontal bars represent the median of occlusion and bleeding times for each group of animals (*n* = 10). ^∗∗∗^*P* < 0.001 versus vehicle control and ^#^*P* < 0.05 and ^###^*P* < 0.001 between two groups after ANOVA and Turkey’s test.

We also evaluated the effect of HRT on FeCl_3_-induced carotid artery thrombosis. The median time to blood flow occlusion induced by FeCl_3_ on carotid artery was significantly prolonged with the oral administration of 100 mg/kg HRT for 1 and 7 days compared to the control (Figures 6A,B). However, blood counts did not differ among the three groups (Table 2). Although the oral administration of HRT (100 mg/kg) for 1 day did not increase the prolongation blood flow occlusion significantly compared to that of the positive control (ASA, 50 mg/kg) (Figure 6A), the oral administration of HRT for 7 days showed a blood flow prolongation equivalent to that induced by ASA (Figure 6B). We further examined whether the oral administration of HRT influences hemostatic function. Tail bleeding time was measured by the cessation of bleeding after tail amputation. We observed no statistically significant differences in tail bleeding time between the mice that received the oral administration of 100 mg/kg HRT for 1 and 7 days and the control mice (Figures 6C,D). However, the oral administration of 50 mg/kg ASA for 1 and 7 days caused a much longer bleeding time than that displayed in the HRT-treated mice and the controls (Figures 6C,D). These results suggest that HRT plays an important role in arterial thrombosis *in vivo* but not in hemostasis.

**Table 2:** The number of circulating blood cells in vehicle and HRT treated mice.

(10^3^/μL)	WBC	NE	LY	MO	RBC	PLT	MPV
Vehicle	4.2 ± 1.3	0.6 ± 0.1	3.3 ± 1.7	0.1 ± 0.0	9.0 ± 1.2	1148 ± 168	7.9 ± 1.6
HRT (1 day)	3.8 ± 1.2	0.7 ± 0.2	2.8 ± 1.5	0.1 ± 0.0	9.0 ± 1.5	1154 ± 159	8.3 ± 0.3
HRT (7 day)	4.1 ± 1.5	0.5 ± 0.1	2.9 ± 1.6	0.1 ± 0.0	9.1 ± 1.0	1139 ± 187	7.4 ± 0.8

*Blood cells from oral administration of vehicle and/or HRT mice were counted using an automated hematology analyzer (ADVIA 2120i, Siemens AG, Germany). Data represent the mean ± SD (*n* = 5 mice per group). WBCs, white blood cells; NEs, neutrophils; LYs, lymphocytes; MO, monocytes; RBCs, red blood cells; PLTs, platelets; MPV, mean platelet volume.*

## Discussion

Although clinical treatment with antiplatelet drugs has been widely used to prevent arterial thrombosis, unwanted bleeding is the primary problem caused by currently used antiplatelet drugs (Eikelboom et al., 2012). Therefore, it would be a great ideal if we could control the targets for developing thrombosis without bleeding effects. Previous studies have shown that traditional plants have an abundant source of novel pharmacologically active compounds (Choi et al., 2015; Yuan et al., 2016). In particular, the antiplatelet activities found in traditional plants have received considerable attention because of their safety and wide range of biological activities (Hladovec, 1972). The current study demonstrated that HRT treatment clearly elicited antiplatelet and antithrombotic activities in mice. Importantly, we have determined that HRT regulates the phosphorylation of PLC and AKT in response to thrombin and CRP stimulation, which thereby regulates granule secretion, TXB_2_ generation, Ca^2+^ mobilization, and aggregation. Thus, this study highlights the important role of HRT in platelet activation and thrombus formation.

According to the previous theory of traditional medicine, HRT is clinically used for anti-inflammatory purposes and to treat acute liver injury without adverse effects (Lee et al., 2014). In recent years, several studies also showed a potential therapeutic effect of HRT in cerebral ischemia (Xu et al., 2000; Zhang et al., 2009; Zhu et al., 2013). Thus, HRT plays multiple roles in preventing several diseases because of its four different herbs that contain a relatively high proportion of polyphenolic compounds, particularly flavonoids; these herbs may be responsible for its pharmacological properties. Notably, flavonoids have received attention in medicinal use because of their ability to reduce cardiovascular risks. Several studies have shown that certain dietary flavonoids inhibit platelet activation and aggregation *in vivo* and *in vitro* (Landolfi et al., 1984; Janssen et al., 1998; Keevil et al., 2000). Furthermore, the diverse mechanisms of flavonoids are displayed through their involvement in different signal transduction pathways, including the inhibition of Ca^2+^ influx, cyclooxygenase and lipoxygenases, cyclic Adenosine monophosphate (cAMP), phosphodiesterase (PDE), tyrosine kinases, PLC, and phosphatidylserine exposure (Landolfi et al., 1984; Akiyama et al., 1987; Polette et al., 1996; Lindahl and Tagesson, 1997; Bucki et al., 2003; El Haouari and Rosado, 2011). However, the mechanisms underlying the actions of flavonoids in platelet functions are not fully understood.

**FIGURE 7:**
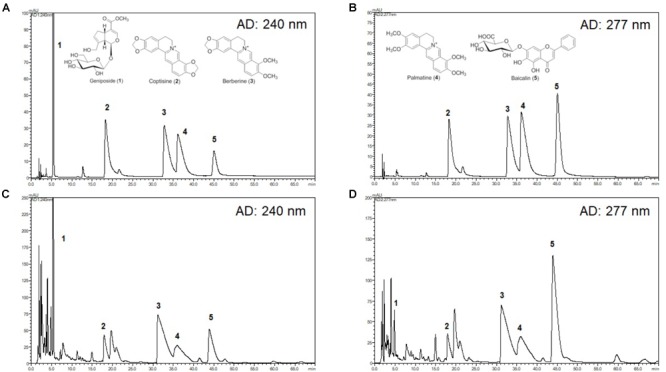
High-performance liquid chromatography (HPLC) chromatograms of HRT. Representative HPLC chromatograms of the standard mixture of five compounds detected at 240 nm **(A)** and 277 nm **(B)**, and HRT at 240 nm **(C)**, and 277 nm **(D)**. Geniposide (1); coptisine (2); palmatine (3); berberine (4); baicalin (5).

Although the mechanism by which HRT regulates platelet responses to all agonists remains unclear, it is likely a result of the different key components of HRT. In this study, we simultaneously analyzed the five compounds in HRT (geniposide, coptisine, berberine, palmatine, and baicalin) using a HPLC (Figure 7). Previously, some studies reported that the main components of HRT displayed antiplatelet activity by suppressing platelet aggregation via the inhibition of PLA2 activity and TXB_2_ production (Hattori et al., 1992; Suzuki et al., 2001; Huang et al., 2002; Zeng et al., 2009; Zhu et al., 2013; Ku and Bae, 2014; Lee et al., 2015; Ma et al., 2015; Seo and Shin, 2015; Wang et al., 2017). Thus, we speculated that most polyphenolic compounds may play an important role in platelet function via regulating cyclooxygenase activity and/or TXB_2_ synthase. Indeed, this speculation is supported by our findings that HRT significantly diminished TXB_2_ production in a concentration-dependent manner (Figures 2C,D). Although the effect of the individual components of HRT is not fully understood, the synergy and additive effects of the individual components and the traditional formula of HRT plays an important role in platelet activation and aggregation. However, to study the pharmacological action of HRT and the potential interactions of different targets, further understanding of the pharmacokinetics and efficacy of the key components of HRT is necessary.

Although the different platelet agonists and adhesive proteins activate platelets through their own receptor signals, the divergent signaling pathways converge into common signaling pathways, which intensify platelet responses (Estevez and Du, 2017). PLC is a major convergent point in platelet signaling pathways. Human platelets predominantly express three different PLC family members, including PLCγ2, PLCβ2, and PLCβ3 (Lee et al., 1996). In particular, PLCβ and PLCγ are activated via Gα_q_- and GPVI-coupled agonists, respectively (Rhee, 2001). Subsequently, activated PLC catalyzes the hydrolysis of phosphatidylinositol 4,5-bisphosphate (PIP_2_) into diacylglycerol (DAG) and inositol 1,4,5-trisphosphate (IP3), which results in the upregulation of PI3K/AKT signaling and the elevation of Ca^2+^ mobilization (Rhee, 2001; Varga-Szabo et al., 2009). It was previously shown that kinase and intracellular Ca^2+^ elevation reciprocally influence one another during platelet activation (Braun et al., 2011; Lang et al., 2013; Moroi and Watson, 2015). In this study, we also observed the elevation of Ca^2+^ mobilization and the phosphorylation of PLCβ3-AKT and PLCγ2-AKT following thrombin and CRP stimulation, respectively. Moreover, we noted that the elevation of intracellular Ca^2+^ mobilization is significantly abrogated in HRT-treated platelets. These results highlight the contribution of HRT to the PLC-AKT signaling pathways via Gα_q_- and GPVI-mediated signaling, thereby regulating platelet aggregation and thrombus formation.

In the *in vivo* study that investigated the effect of orally administered HRT, we examined FeCl_3_-induced *in vivo* thrombosis and bleeding time assay. We found that the orally administration of HRT is critical for FeCl_3_-induced *in vivo* thrombus formation. In addition, because FeCl_3_-induced injury can disrupt the endothelium (Kurz et al., 1990), our finding further suggests that HRT can regulate endothelial cells in arterial thrombosis. Importantly, the administration of HRT in mice did not impair hemostatic functions at the site of tail transaction. Because tail bleeding time may not be a reliable analysis of platelet contribution to hemostatic function, we also observed no increase in bleeding from the surgery site during the FeCl_3_-induced injury arterial thrombosis study. Thus, these findings support that HRT is important for regulating platelet function but is not essential for hemostasis in mice.

## Conclusion

Our results have defined the importance of HRT in platelet activation and thrombus formation without adverse effects when compared with ASA. Therefore, HRT may have therapeutic potential for preventing thrombotic diseases. Future studies on HRT in human platelets will be extremely helpful for further understanding of the role of HRT during thrombosis and hemostasis.

## Author Contributions

KK designed and performed the research, collected and analyzed the data, and wrote the manuscript. HD, TO, and K-YK performed the research and provided the important data. TK provided the important data and wrote the manuscript. JM provided the data. K-IP initiated and designed the research, analyzed the data, and wrote the manuscript. All authors reviewed the manuscript.

## Conflict of Interest Statement

The authors declare that the research was conducted in the absence of any commercial or financial relationships that could be construed as a potential conflict of interest.
