# Supplementary material for: Antiplatelet and Antithrombotic Activity of a Traditional Medicine, Hwangryunhaedok-Tang
Source: Front Pharmacol. 2019 Jan 9;9:1502. doi: 10.3389/fphar.2018.01502 (PMC6333754; doi:10.3389/fphar.2018.01502)

**SUPPLEMENTAL METHODS**

**Platelet aggregation.** Washed platelets were pre-incubated with 0.01% DMSO or 100 µg/ml HRT for 10 minutes for at 37 °C and then stimulated with thrombin (0.2 U/ml), CRP (2 µg/ml), ADP (30 µM), and U46619 (10 µM). Platelet aggregation was monitored in a platelet aggregometer (Chronolog Corp, Havertown, PA) at 37 °C with stirring (1,000 rpm).

**SUPPLEMENTAL FIGURE LEGENDS**

**Figure S1. HRT does not regulate platelet aggregation induced by a high concentration of thrombin, CRP, ADP, or U46619.** Platelet aggregation of 0.01% DMSO or 100 µg/ml HRT pretreated platelets were induced by stimulation with 0.2 U/ml Thrombin (A), 2 µg/ml CRP (B), 30 µM ADP (C), and 10 µM U46619 (D).

Figure S1


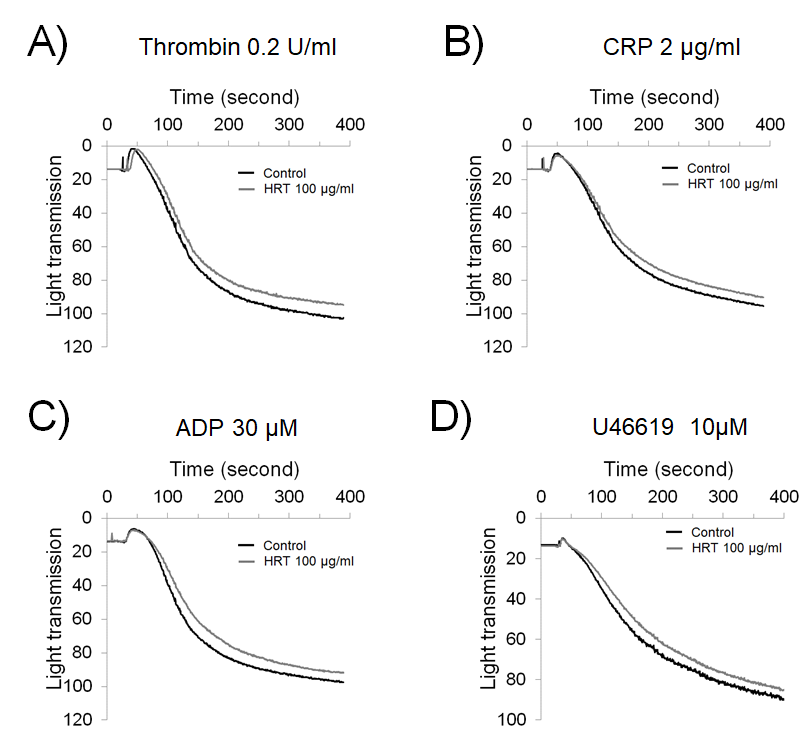

Supplement: Supplementary file 1 [file Table_1.DOCX]
