# Supplementary material for: Antiplatelet and Antithrombotic Activity of a Traditional Medicine, Hwangryunhaedok-Tang
Source: Front Pharmacol. 2019 Jan 9;9:1502. doi: 10.3389/fphar.2018.01502 (PMC6333754; doi:10.3389/fphar.2018.01502)
Supplement: Supplementary file 2 [file Data_Sheet_1.PDF]

## SUPPLEMENTAL METHODS

**Platelet aggregation.** Washed platelets were pre-incubated with 0.01% DMSO or 100 µg/ml HRT for 10 minutes for at 37 °C and then stimulated with thrombin (0.2 U/ml), CRP (2 µg/ml), ADP (30 µM), and U46619 (10 µM). Platelet aggregation was monitored in a platelet aggregometer (Chronolog Corp, Havertown, PA) at 37 °C with stirring (1,000 rpm).

## SUPPLEMENTAL FIGURE LEGENDS

**Figure S1. HRT does not regulate platelet aggregation induced by a high concentration of thrombin, CRP, ADP, or U46619.** Platelet aggregation of 0.01% DMSO or 100 µg/ml HRT pretreated platelets were induced by stimulation with 0.2 U/ml Thrombin (A), 2 µg/ml CRP (B), 30 µM ADP (C), and 10 µM U46619 (D).

# Figure S1

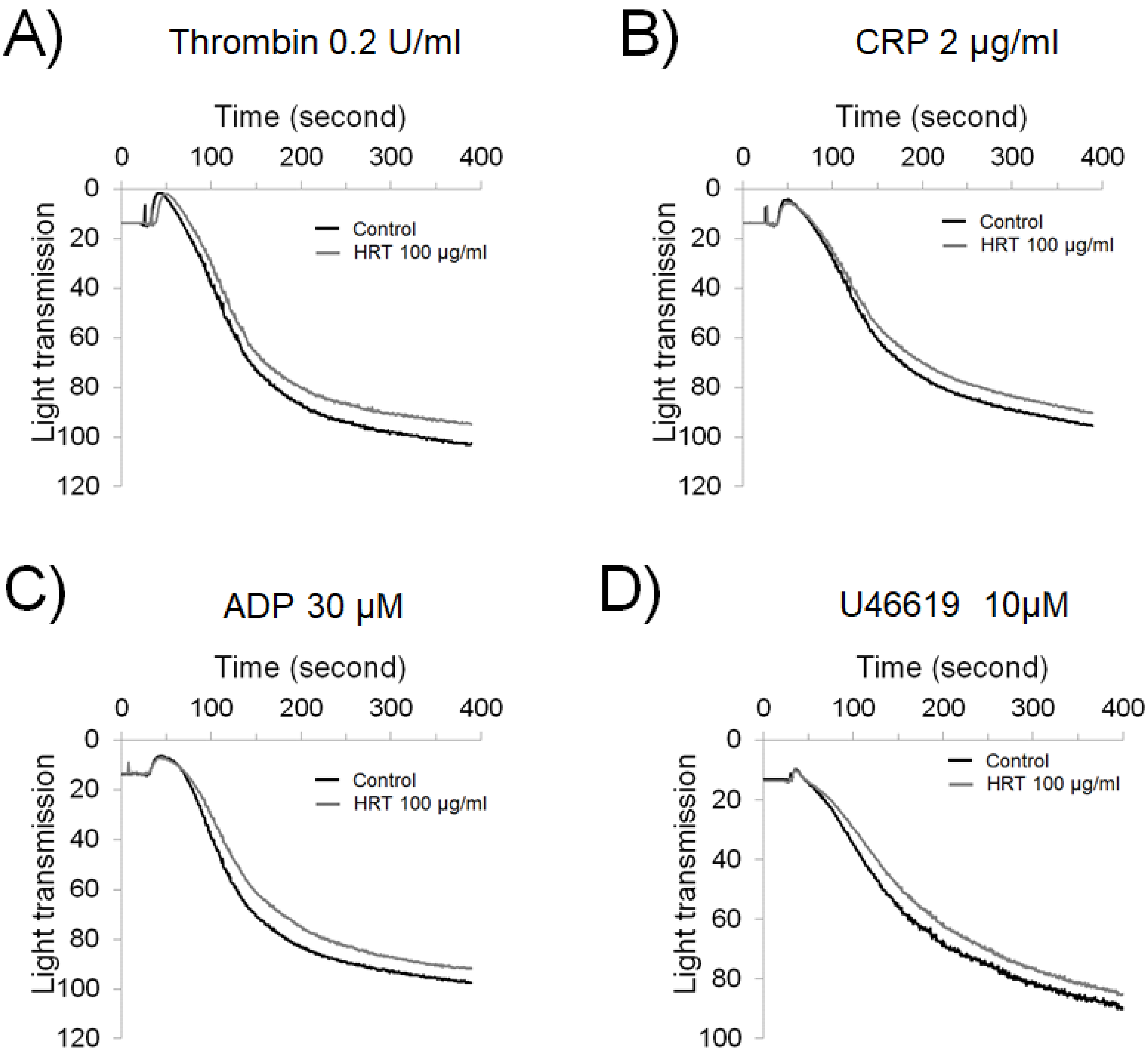

A)
Thrombin 0.2 U/ml
Time (second)
0
100
200
300
400
Light transmission
0
20
40
60
80
100
120
Control
HRT 100 µg/ml
B)
CRP 2 µg/ml
Time (second)
0
100
200
300
400
Light transmission
0
20
40
60
80
100
120
Control
HRT 100 µg/ml
C)
ADP 30 µM
Time (second)
0
100
200
300
400
Light transmission
0
20
40
60
80
100
120
Control
HRT 100 µg/ml
D)
U46619 10µM
Time (second)
0
100
200
300
400
Light transmission
0
20
40
60
80
100
Control
HRT 100 µg/ml
